# Managing the rumen hydrogen economy to improve feed efficiency and climate-smart livestock production

**DOI:** 10.3389/fvets.2026.1843670

**Published:** 2026-05-29

**Authors:** Onesimus Kiadii, Longping Li

**Affiliations:** 1 College of Advanced Agricultural Sciences, Yulin University, Yulin City, China; 2 Shaanxi Provincial Engineering and Technology Research Center of Cashmere Goats, Yulin University, Yulin City, China

**Keywords:** climate-smart livestock, enteric methane, feed efficiency, fermentation kinetics, methane mitigation, rumen hydrogen economy

## Abstract

Enteric methane (CH₄) emissions from ruminant livestock present dual challenges for agricultural sustainability: contributing to greenhouse gas emissions while reducing feed conversion efficiency and animal productivity. Methane is produced by methanogenic archaea utilizing metabolic hydrogen (H₂) generated during ruminal fermentation. This hydrogen economy is central to fermentation efficiency, nutrient utilization, and methane formation. Conventional mitigation strategies have primarily focused on inhibiting methanogenesis; however, these approaches often yield inconsistent results across production systems and lack an integrative framework for systematic application. This narrative review proposes a shift in perspective from methane suppression to the management of H₂ flow within the rumen hydrogen economy and introduces two complementary conceptual frameworks to guide this approach. The genetic-microbiome co-evolution framework conceptualizes the rumen microbiome as a partially heritable trait shaped by host genetic and environmental selection, providing a theoretical basis for selecting low-emission, feed-efficient animals. The conceptual fermentation kinetics framework provides a mechanistic basis for understanding how dietary inputs and microbial interactions influence the distribution of hydrogen among competing metabolic pathways, including methanogenesis and propionate formation. Together, these frameworks establish a systems-level perspective that may inform the development of integrated strategies combining host genetic selection, precision nutrition, and microbial management. While substantial validation remains necessary, this approach provides a conceptual foundation for advancing methane mitigation from descriptive observation toward mechanistic interpretation, with the ultimate goal of supporting climate-smart livestock production systems.

## 1 Introduction

Ruminant livestock represent a significant source of anthropogenic methane (CH₄), a potent greenhouse gas with important implications for climate change mitigation and agricultural sustainability (1). Methane originates predominantly from the rumen, a complex anaerobic microbial ecosystem in which fermentation governs both enteric CH₄ production and feed utilization efficiency (2). Beyond its environmental impact, CH₄ formation represents a loss of dietary energy, estimated at 2–12% of gross energy intake (3) that could otherwise support animal growth, milk production, and reproductive function. Consequently, developing strategies that simultaneously improve feed efficiency and reduce CH₄ intensity has become a central priority for sustainable ruminant agriculture, with global targets calling for reductions of approximately 30% in daily CH₄ output without compromising animal performance (4).

Given that methane formation is an inherent outcome of microbial fermentation, effective mitigation depends on understanding and managing the rumen hydrogen economy, the interconnected network of microbial processes governing hydrogen production, transfer, and utilization during fermentation (5). From an agricultural systems perspective, this hydrogen network represents a metabolic juncture where nutrition, genetics, and management decisions can influence both environmental performance and production efficiency (6).

Despite extensive research, current methane mitigation strategies remain fragmented and often yield inconsistent results across livestock systems (7, 8). Traditional approaches have focused largely focused on standalone interventions, such as dietary adjustments or chemical inhibitors, and have lacked a comprehensive conceptual framework to guide their systematic application. This has contributed to conflicting findings, including the “operationalize,” in which similar nutritional strategies produce divergent methane responses across studies because unaccounted variation in hydrogen partitioning dynamics is not considered (9). Although advances in metagenomics and other omics technologies have substantially enhanced understanding of rumen microbial composition and function (10, 11), descriptive insights alone cannot reliably predict system-level behavior or support consistent management practices (12). Methane production is fundamentally driven by metabolic hydrogen (H₂) generated during fermentation; however, most mitigation strategies treat methane as an endpoint rather than addressing the hydrogen dynamics that regulate the rumen hydrogen economy (13).

**Figure 1:**
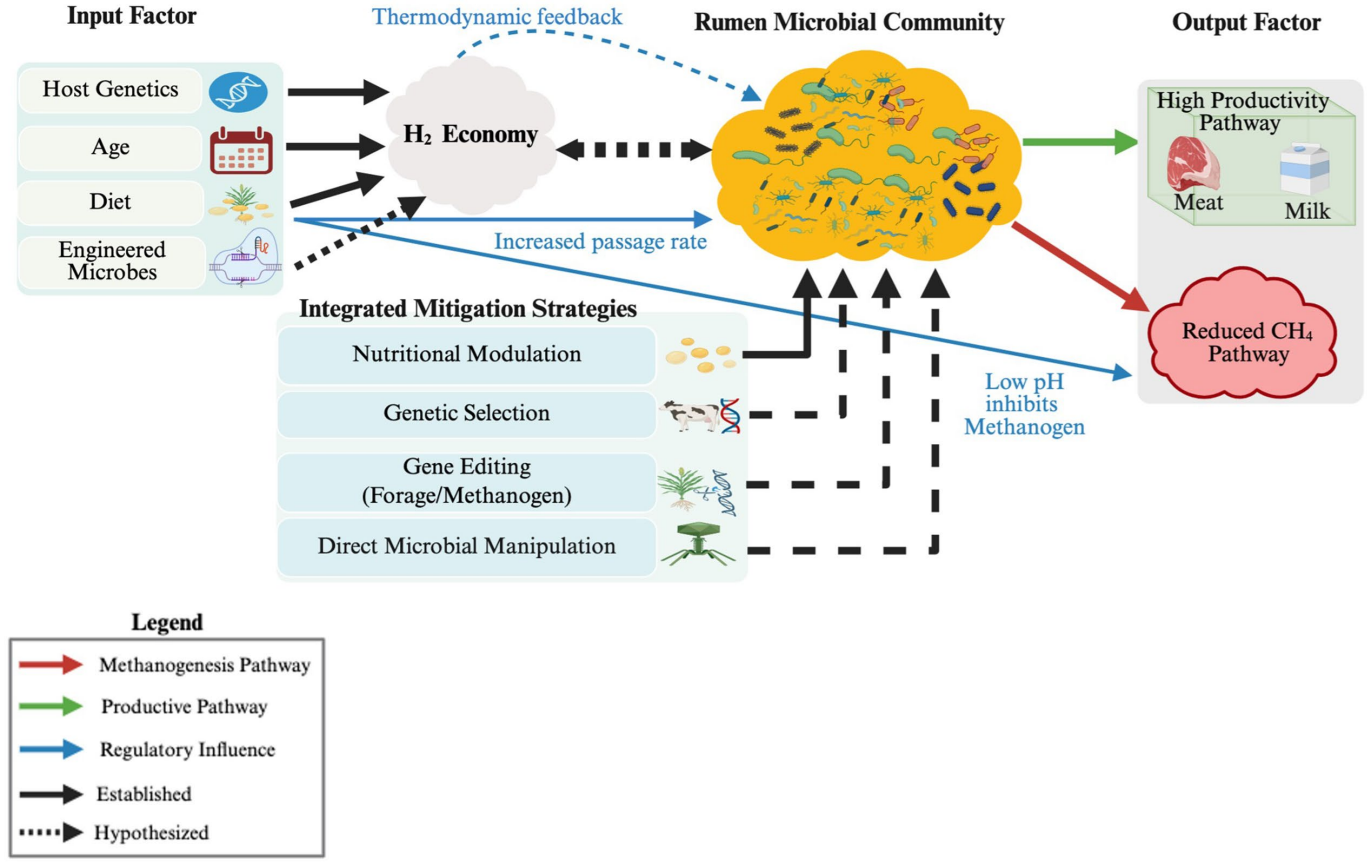
Systems-level overview of ruminal hydrogen flow. Schematic showing how dietary, microbial, and genetic factors influence hydrogen partitioning among competing metabolic pathways. Solid arrows indicate established hydrogen flows with strong empirical support; dashed arrows indicate inferred or hypothesized interactions. Green pathways represent productive hydrogen sinks (e.g., propionate formation). Red pathways represent methanogenesis. Blue arrows indicate regulatory influences (e.g., low-pH inhibition, passage-rate effects, and thermodynamic feedback via H2 partial pressure) that modulate flux through the network. Created in BioRender. Kiadii O. (2026) https://BioRender.com/l6m9zev.

This narrative review addresses this gap by synthesizing evidence from host genetics, microbial ecology, and fermentation biochemistry into a unified conceptual framework centered on hydrogen flow. Two complementary frameworks are proposed: (1) the Genetic-Microbiome Co-evolution Framework, which posits that the rumen microbiome exhibits partial heritability influenced by host genetic variation, thereby providing a theoretical basis for selecting animals predisposed to low-emission, feed-efficient fermentation profiles (14, 15); and (2) the Conceptual Fermentation Kinetics Framework, which provides a mechanistic basis for understanding how dietary inputs and microbial interactions influence hydrogen flux among competing metabolic pathways (9, 16, 17). Importantly, these frameworks are presented as hypothesis-generating conceptual tools rather than validated predictive models, reflecting the current state of knowledge and the need for further empirical validation. Together, they establish a systems-level perspective of the rumen hydrogen economy (Figure 1) and offer a foundation for developing integrated mitigation strategies that may improve both environmental and productive outcomes in ruminant production systems. A systems-level overview of the rumen hydrogen economy, illustrating the conceptual integration of dietary, genetic, and microbial influences on hydrogen partitioning, is presented in Figure 1.

## 2 Review methodology

### 2.1 Approach and scope

This review employs a narrative synthesis approach with structured literature selection elements. The objective was to integrate evidence from ruminant nutrition, microbial ecology, host genetics, and fermentation biochemistry to develop conceptual frameworks for managing the rumen hydrogen economy. A narrative review format was selected as appropriate for synthesizing diverse evidence streams and proposing conceptual advances, while structured search and selection criteria were applied to enhance transparency and reproducibility (18).

### 2.2 Literature search strategy

Literature searches were conducted between September 2025 and January 2026 using the following electronic databases: Web of Science, Scopus, PubMed, and Google Scholar. Search strategies combined terms from three conceptual domains using Boolean operators:

Domain 1 (Rumen fermentation): “rumen fermentation,” “ruminal metabolism,” “hydrogen metabolism,” “methanogenesis,” “volatile fatty acids.”

Domain 2 (Microbiome and genetics): “rumen microbiome,” “rumen microbiota,” “methanogenic archaea,” “host genetics,” “heritability,” “genetic selection.”

Domain 3 (Mitigation): “methane mitigation,” “enteric methane,” “feed additives,” “3-nitrooxypropanol,” “dietary intervention,” “climate-smart livestock.”

Searches were limited to English-language publications. Reference lists of identified articles and relevant reviews were manually screened to identify additional sources.

### 2.3 Inclusion and exclusion criteria

Studies were included if they met the following criteria: (1) addressed rumen methanogenesis, hydrogen metabolism, or methane mitigation strategies in ruminant livestock; (2) were published in peer-reviewed journals; (3) were published between 2000 and 2026, with foundational studies before 2000 included where essential for context. Both primary research (experimental, observational, and modeling studies) and secondary research (reviews and meta-analyses) were considered.

Studies were excluded if they: (1) focused exclusively on non-ruminant systems; (2) addressed manure management emissions without rumen fermentation context; (3) were published only as conference abstracts, editorials, or non-peer-reviewed materials.

### 2.4 Synthesis approach

Identified literature was organized thematically around the concept of the rumen hydrogen economy. Evidence was synthesized to address: (1) factors shaping rumen microbial community structure and function (diet, host genetics, age); (2) hydrogen production and consumption pathways; (3) intervention strategies and their mechanistic basis; and (4) integration into conceptual frameworks. Critical appraisal of evidence quality and consistency was conducted throughout, with particular attention to conflicting findings and variability across production systems.

### 2.5 Limitations

As a narrative review, this synthesis does not employ formal meta-analytic methods or quantitative evidence grading. The proposed conceptual frameworks are presented as hypothesis-generating tools requiring further empirical validation. Quantitative estimates cited from individual studies should be interpreted within their specific experimental contexts and may not generalize across all production systems, species, or management conditions.

## 3 The rumen microbial ecosystem and hydrogen economy

### 3.1 Initiation of fermentation: the primary role of bacteria and fungi

Rumen fermentation is initiated by a consortium of anaerobic fungi and bacteria that collectively degrade complex plant structural carbohydrates (19). Anaerobic fungi physically penetrate lignified tissues, increasing cellulose accessibility, while specialized cellulolytic bacteria, including *Ruminococcus* and *Fibrobacter,* hydrolyze structural carbohydrates into fermentable sugars (20, 21). These sugars are subsequently converted into volatile fatty acids (VFAs), primarily acetate, propionate, and butyrate, which supply up to 70% of the host’s metabolizable energy (22). This fermentative process represents the primary source of metabolic hydrogen (H₂) that drives the rumen hydrogen economy.

Notably, shifts in bacterial community composition can redirect H₂ flux toward alternative metabolic sinks. For example, in sheep, supplementation with bio-fermented rice straw increased the abundance of *Prevotella* spp. and enhanced propionate production, a hydrogen-consuming pathway resulting in reduced methane yield (23). This illustrates that primary fermentation is not merely an energy-generating process but also the first control point of the ruminal hydrogen economy.

### 3.2 Hydrogen economy and the central role of methanogenic archaea

**Figure 2:**
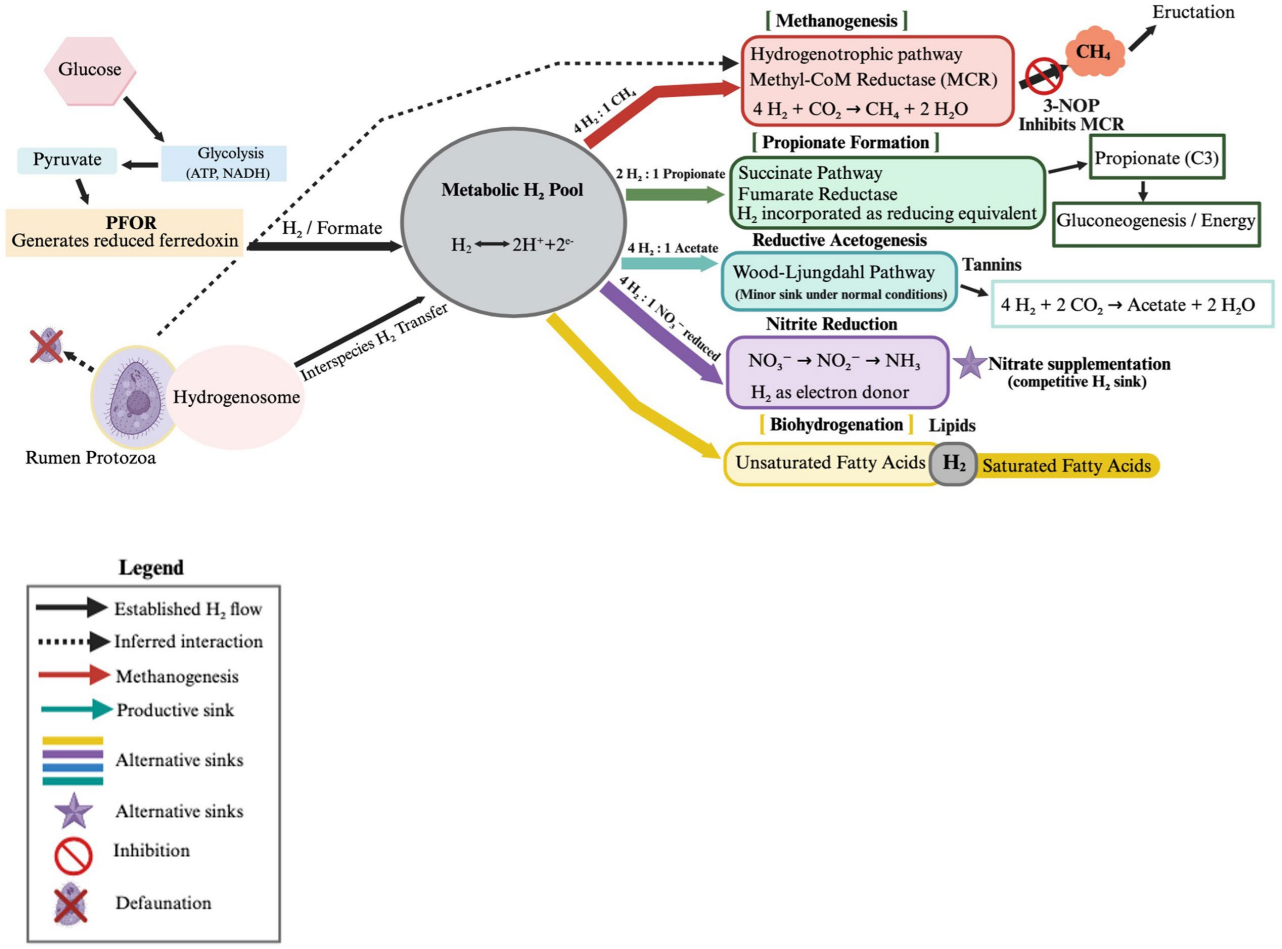
Core metabolic pathways driving rumen methanogenesis. Detailed representation of hydrogen-producing and hydrogen-consuming pathways in the rumen. Left: Primary H2 sources include glycolysis coupled to pyruvate: ferredoxin oxidoreductase (PFOR) and hydrogenosomes within protozoa. Center: Metabolic H2 pool interconverts with formate. Right: Competing H2 sinks include methanogenesis (red), propionate formation (green), reductive acetogenesis (teal), nitrate/nitrite reduction (purple), and biohydrogenation (yellow). Stoichiometric relationships are indicated adjacent to arrows. Intervention points for major mitigation strategies (3-NOP, lipids, tannins, nitrate, defaunation) are marked. Solid arrows indicate established H2 flows; dashed arrows indicate inferred interactions such as physical association between protozoa and methanogens. Created in BioRender. Kiadii, O. (2026) https://BioRender.com/y4vecnd.

Hydrogen (H₂) generated during ruminal fermentation occupies a central position in the microbial energy network and is competitively partitioned between methanogenic and alternative pathways (Figure 2). The H₂ released by fermentative bacteria and fungi must be efficiently scavenged to prevent its accumulation, which would otherwise inhibit key redox-sensitive enzymatic reactions, particularly the reoxidation of reduced cofactors (NADH, FADH₂), and impair overall rumen function (5). Methanogenic archaea, dominated by members of the genus *Methanobrevibacter*, serve as the primary hydrogen sink by coupling H₂ oxidation with CO₂ reduction to produce methane via the hydrogenotrophic pathway (24, 25). As terminal electron acceptors, methanogens stabilize fermentation while generating methane, which represents both an energetic loss to the host and a significant source of greenhouse gas emissions. Although acetoclastic and methylotrophic methanogenesis also occur, hydrogenotrophic pathways predominate in the rumen due to the high availability of H₂ as a substrate (5). The core metabolic pathways responsible for hydrogen production and consumption within the rumen, including the central role of methanogenic archaea and competing hydrogen sinks, are depicted in Figure 2.

The abundance and activity of methanogenic archaea are dynamic rather than static. Host genetic factors exert a measurable influence on methanogen population size and transcriptional activity, with heritable variations linked to specific chromosomal regions (14). In beef cattle, Roehe et al. (14) reported that sire progeny groups differed significantly in methane emissions (range: 136–205 g/day, measured under controlled feeding conditions) and rumen microbial composition, indicating the presence of heritable traits associated with methane production. Quantitative analyses from this study revealed that diversity indices, the relative abundance of approximately 34% of microbial taxa examined (59 of 174), and total bacterial copy number exhibited heritability estimates (h^2^) ≥ 0.15, suggesting moderate heritability influenced by additive host genetic effects in this population.

The ratio of archaea to bacteria in rumen content, a key factor associated with methane production, showed consistent ranking across sire groups and correlated with methane emissions (*r* = 0.80 for g/day; *r* = 0.65 for g/kg dry matter intake). This association suggests host genetic influence on methanogenic archaea such as *Methanobrevibacter*, though causation cannot be definitively established from these observational data. Genome-wide association studies identified 19 bovine single-nucleotide polymorphisms (SNPs) across 12 chromosomes significantly associated with the abundance of 14 heritable rumen microbial taxa, providing evidence that specific host genomic regions correlate with rumen microbiome composition (14, 15). Consistent with these findings, Ben Shabat et al. (26) demonstrated that microbiome-dependent mechanisms contribute to differences in energy-harvesting efficiency among ruminants, with more efficient animals exhibiting fermentation profiles that produce less methane as a metabolic byproduct.

Animal age further modulates this system. Younger ruminants typically exhibit lower methane emissions owing to the incomplete establishment of methanogenic communities and altered hydrogen flux patterns (27, 28). Studies on dairy buffaloes have demonstrated that age significantly affects rumen microbial (27, 28). Although *Bacteroidetes* and *Firmicutes* consistently dominate the rumen microbiome across developmental stages, their relative abundances shift. Younger ruminants (e.g., one-year-old buffaloes) exhibit higher abundance of *Lactobacillus* and *Prevotella*, while older animals show increased levels of *Bacteroides* and methanogenic archaea, particularly *Methanobrevibacter* (28). These compositional changes are accompanied by functional shifts in microbial metabolism; younger ruminants demonstrate greater activity in carbohydrate metabolism pathways, while older animals show enhanced protein metabolism and DNA repair activity (28).

### 3.3 Integrated microbial synergy and functional outcomes

The emergent properties of this microbial consortium extend beyond methane emissions to directly affect animal productivity and product quality. Microbiome-driven shifts that decrease methane production often coincide with altered nutrient partitioning. In sheep fed bio-fermented rice straw, microbial restructuring was associated with systemic metabolic changes, including increased bile acid biosynthesis and unsaturated fatty acid metabolism, leading to improved meat quality with higher monounsaturated fatty acid (MUFA) content (23). Similarly, in dairy cattle, the relative abundance of key microbial groups, including the *Firmicutes*-to-*Bacteroidetes* ratio and specific taxa such as *Prevotella* and *Lachnospiraceae,* is correlated with milk fat and protein yields (22). These associations (noting that correlation does not establish causation) suggest that the rumen microbiome functions as a regulatory interface connecting fermentation efficiency, hydrogen management, methane emissions, and production traits.

### 3.4 Key factors shaping the microbiome and methanogenesis

Diet, host genetics, and age do not independently drive methane emissions; they influence a common control point: the production, allocation, and consumption of metabolic hydrogen (H₂) within the rumen. The associations among these drivers, rumen microbial community composition, fermentation pathway distribution, and hydrogen flux are summarized in Table 1. It is important to note that the relationships presented represent predominant patterns observed under typical production conditions; exceptions and context-dependent variations occur, and the quantitative nature of these relationships requires further characterization across diverse systems.

**Table 1:** Associations of diet, host genetics, and age with rumen microbial structure, hydrogen Metabolism, and enteric CH₄ production.

Driver	Observed microbial shifts	Directional effect on H₂/CH₄	Observed production outcomes	Key references
Diet				
High-forage	↑ *Fibrobacter*, *Ruminococcus* (fiber degraders)	↑ CH₄ (sustained H₂ production)	Variable digestibility; lower intake potential	(21, 23)
High-concentrate	↑ Amylolytic bacteria; ↓ Fiber degraders	↓ CH₄ yield (shift to propionate)	↑ Milk yield; ↑ Growth rate; Acidosis risk	(7, 22)
Bio-fermented straw	↑ *Prevotella* spp.	↓ CH₄ (H₂ diverted to propionate)	↑ Body fat; ↑ Digestibility; ↑ Growth rate	(23)
Lipid supplementation	↓ Protozoa; ↓ Methanogens	↓ CH₄ (inhibition + H₂ sink competition)	May ↓ DMI at high inclusion; ↓ Milk fat	(35, 36)
Host genetics				
Sire genetic groups	Heritable variation in *Methanobrevibacter* abundance	Variation in CH₄ (136–205 g/d range in studied population)	86% of feed efficiency variation associated with microbial factors	(26, 36)
Archaea: bacteria ratio	Stable ranking across genetic lines; correlated with CH₄ (*r* = 0.80 g/d)	↑ CH₄ with ↑ ratio	Associated with feed conversion efficiency	(14)
Genomic associations	19 SNPs across 12 chromosomes associated with microbial taxa	Host genotype correlates with microbiome composition	Potential selection target	(14, 15)
Age				
Young ruminants	↑ *Lactobacillus*, *Prevotella*	↓ CH₄ (immature methanogen communities)	↑ Starch/sucrose degradation capacity	(27, 28)
Older ruminants	↑ *Bacteroides*, *Methanobrevibacter*	↑ CH₄ (established methanogens)	↑ Protein utilization; ↑ Feed efficiency	(27)

Relationships presented are associative patterns observed under typical production conditions. Magnitudes vary across studies, species, and management contexts. Directional arrows (↑ increase, ↓ decrease) indicate predominant trends rather than universal relationships.

This synthesis demonstrates that dietary composition, host genetic background, and animal age are associated with CH₄ emissions primarily through their influence on H₂ production during fermentation and its allocation among different microbial sinks. Rather than operating through entirely separate mechanisms, these factors affect fermentation kinetics, microbial interactions, and terminal electron-accepting pathways. This synthesis provides the empirical foundation for the conceptual frameworks introduced in Section 4, which consider H₂ flow as the central organizing principle for understanding CH₄ output.

**Figure 3:**
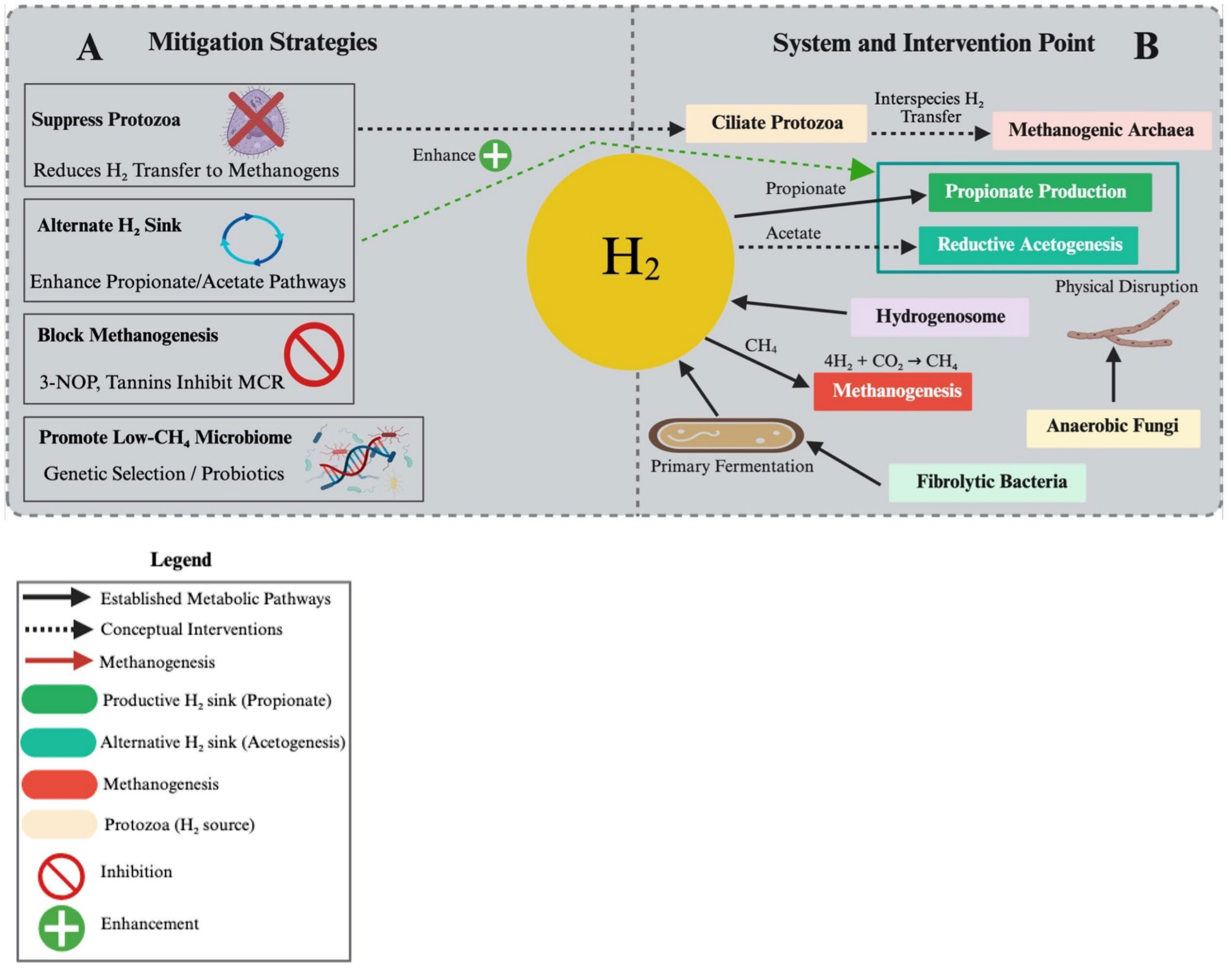
Schematic representation of the ruminal microbial hydrogen network and potential intervention points. **(A)** Major mitigation strategies targeting rumen methanogenesis. **(B)** Microbial groups involved in hydrogen (H2) production and utilization during ruminal fermentation, including fibrolytic bacteria, anaerobic fungi, ciliate protozoa (with hydrogenosomes), and methanogenic archaea. Primary fermentation generates H2, which is partitioned among competing sinks: methanogenesis (red), propionate production (green), and reductive acetogenesis (teal). Solid arrows indicate established metabolic pathways with strong empirical support. Dashed arrows indicate inferred interactions (e.g., interspecies H2 transfer between protozoa and methanogens) or conceptual intervention targets. Intervention points for suppression, alternative sink enhancement, and methanogenesis inhibition are indicated. Created in BioRender. Kiadii, O. (2026) https://BioRender.com/y4vecnd.

The interconnected microbial hydrogen network and the principal intervention points through which dietary, microbial, and management-based strategies may influence hydrogen flux are summarized schematically in Figure 3.

## 4 Conceptual frameworks for managing the rumen hydrogen economy

### 4.1 Framework 1: genetic-microbiome co-evolution

The Genetic-Microbiome Co-evolution Framework conceptualizes the rumen microbiome as partially heritable and potentially selectable, influenced by, though not fully determined by, host genetic variation. This framework extends beyond previous observations of host-genetic effects on individual microbial taxa by proposing that the rumen microbiome can be systematically considered in genetic improvement programs (14, 15).

#### 4.1.1 Empirical foundations

Several lines of evidence support the plausibility of this framework, though each requires careful contextual interpretation. Roehe et al. (14) reported significant differences in methane output and microbial composition among sire groups in beef cattle, suggesting host genetic influence on methanogenic capacity in the population studied. The archaeal-to-bacterial ratio, which is correlated with methane production in this dataset, showed consistent ranking across genetic lines, suggesting host regulation of methanogen abundance. Genome-wide association studies have identified bovine SNPs associated with the relative abundance of specific microbial taxa, including *Methanobrevibacter* (14, 15). Importantly, these associations are statistical in nature and do not establish direct causal relationships between host genotype and microbial phenotype.

Functionally, variation in key microbial genes involved in methanogenesis (e.g., *mcrA*, encoding methyl-coenzyme M reductase) has been associated with variation in methane emissions, though the strength of this association varies across studies and populations (11, 29). Modeling approaches suggest that incorporating microbial biomarkers into genomic selection programs may improve the accuracy of identifying low-emission animals by approximately 34% in the populations studied, though this estimate derives from specific modeling assumptions and requires validation in diverse production settings (11).

#### 4.1.2 Conceptual pillars

This framework rests on three mechanistic propositions requiring further empirical validation: (i) host genomic regions correlate with the abundance of specific microbial taxa, though causal mechanisms remain to be elucidated; (ii) microbial functional gene abundance links microbiome composition to the methane phenotype, with the strength of this association varying across contexts; and (iii) microbial biomarkers may inform genetic selection programs, though practical implementation faces substantial challenges including genotype-by-environment interactions and the cost of microbiome profiling.

#### 4.1.3 Limitations and uncertainties

Several important limitations must be acknowledged. Heritability estimates vary substantially across populations, environments, and measurement methods (30). The mechanisms by which host genotype influences microbial community assembly remain incompletely characterized and likely involve complex interactions among immune function, gut anatomy, digesta passage rate, and salivary composition (31). Moreover, genotype-by-environment interactions may substantially influence the realized response to selection, and the economic feasibility of microbiome-informed breeding programs has not been established (32). Selection based on microbial biomarkers, therefore, represents a promising but still developing approach requiring substantial further research before practical implementation.

### 4.2 Framework 2: conceptual fermentation kinetics framework

While the genetic-microbiome co-evolution framework addresses longer-term selection strategies, the conceptual fermentation kinetics framework provides a mechanistic basis for understanding how dietary and management factors influence methane formation in production systems. This framework connects CH₄ output to the dynamic balance between H₂ production and consumption within the rumen, drawing on principles of microbial growth kinetics and mass balance (9, 16, 17).

#### 4.2.1 Conceptual basis

Methane formation within the rumen hydrogen economy can be conceptualized as a function of the balance between hydrogen production and its allocation among competing metabolic pathways. For heuristic purposes, this relationship may be represented conceptually as: $R_{{\mathrm{CH}}_{4}}=k_{m}(R_{\text{prod}}-\sum R_{\text{sink},i})$ , where $R_{\text{prod}}$ represents the rate of hydrogen generation during microbial fermentation, $\sum R_{\text{sink},i}$ represents the cumulative rate of hydrogen consumption by alternative pathways (propionate formation, reductive acetogenesis, nitrate reduction, and biohydrogenation), and $k_{m}$ reflects the efficiency of methanogenic hydrogen capture. This is a conceptual mass-balance framework intended for heuristic interpretation rather than quantitative prediction. The terms cannot currently be measured directly *in vivo* and are presented to illustrate the principle that methane production reflects the net outcome of hydrogen partitioning across the rumen microbial network.

#### 4.2.2 Biological factors influencing framework components

The relationship described conceptually above is modulated by multiple interacting biological factors (5, 13):

- Hydrogen partial pressure ( $P_{H_{2}}$ ): Thermodynamic constraints determine which metabolic pathways are energetically favorable at a given $P_{H_{2}}$ . Methanogenesis is thermodynamically favorable across a wide $P_{H_{2}}\;$ range, while alternative pathways, such as reductive acetogenesis, require lower $P_{H_{2}}$ conditions for thermodynamic feasibility.
- Formate dynamics: Formate serves as an alternative electron carrier that can be interconverted with H₂, adding complexity to hydrogen flux measurements and pathway predictions.
- Substrate class: Fermentation of structural carbohydrates (cellulose, hemicellulose) typically yields higher acetate: propionate ratios and greater net H₂ production compared to non-structural carbohydrate fermentation.
- Passage rate: Increased digesta passage rate can reduce methanogen populations through washout, though this effect interacts with methanogen growth rates and substrate availability.
- Ruminal pH: Low pH inhibits methanogen activity and favors propionate-producing bacteria, altering hydrogen sink distribution.
- Intake level: Higher dry matter intake increases absolute H₂ production ( $R_{\text{prod}}$ ) while often decreasing methane yield (g CH₄/kg DMI) due to passage rate effects.
- Microbial adaptation: Rumen microbial communities exhibit functional redundancy and adaptive capacity that may partially compensate for interventions targeting specific pathways.

#### 4.2.3 Limitations of the conceptual framework

This framework has important limitations that must be acknowledged. The variables $R_{\text{prod}}$ , $\sum R_{\text{sink},i}$ , and $k_{m}$ represent aggregated processes that cannot currently be measured directly *in vivo*. The framework simplifies complex microbial interactions and does not account for spatial heterogeneity within the rumen, diurnal variation in fermentation patterns, or individual animal variation in digesta passage behavior. Furthermore, the framework does not incorporate thermodynamic constraints explicitly, nor does it account for formate-mediated electron transfer that may bypass H₂ as an intermediate (5). These limitations underscore that the framework is intended as a conceptual organizing tool rather than a quantitative predictive model.

### 4.3 Application of the conceptual framework to representative scenarios

To illustrate the interpretive utility of this framework, three contrasting scenarios are examined conceptually:

#### 4.3.1 Scenario A: high-forage diet

Fermentation of fiber-rich substrates by cellulolytic bacteria generates substantial H₂ ( $R_{\text{prod}}$ elevated) through acetate-producing pathways. Propionate production is relatively limited ( $\sum R_{\text{sink},i}$ low to moderate), and methanogens efficiently capture available H₂ ( $k_{m}$ high). The net outcome is elevated $R_{{\mathrm{CH}}_{4}}$ per unit of feed intake (33).

#### 4.3.2 Scenario B: high-concentrate diet

Rapid fermentation of non-structural carbohydrates favors amylolytic bacteria and protozoal populations, altering fermentation stoichiometry. Propionate production increases substantially ( $\sum R_{\text{sink},i}$ elevated), competing effectively with methanogens for available H₂. Reduced ruminal pH may directly inhibit methanogen activity ( $k_{m}$ ) by reducing it. The net outcome is typically reduced $R_{{\mathrm{CH}}_{4}}$ per unit of feed, though absolute emissions depend on intake level (7).

#### 4.3.3 Scenario C: 3-nitrooxypropanol (3-NOP) intervention

3-NOP directly inhibits methyl-coenzyme M reductase, the terminal enzyme in methanogenesis, effectively reducing $k_{m}$ without directly altering $R_{\text{prod}}$ . Hydrogen that would otherwise be captured by methanogens is redirected to alternative sinks, particularly propionate formation (i.e., $\sum R_{\text{sink},i}$ increases via mass action). The net outcome is a substantial reduction in $R_{{\mathrm{CH}}_{4}}$ with minimal effects on feed intake or overall fermentation, provided alternative sink capacity is sufficient (11, 29).

These scenarios illustrate how the conceptual framework can organize the interpretation of observed methane responses across different production contexts, though they do not constitute quantitative predictions.

## 5 Mitigation strategies within the hydrogen economic framework

Translating rumen hydrogen economy principles into practical methane reduction requires systematic evaluation of intervention strategies based on their mechanisms of action, evidence maturity, and production system compatibility. Table 2 provides a structured classification of major mitigation strategies across these dimensions.

**Table 2:** Classification of methane mitigation strategies by evidence maturity, readiness level, and practical feasibility.

Strategy	Mechanism of action	Evidence maturity	Practical readiness	Reported CH₄ reduction	Performance risk	Regulatory constraints	Durability concerns
Lipid supplementation	Direct methanogen inhibition; protozoal suppression; biohydrogenation	Established	Deployable	10–25%	Moderate (digestibility, intake)	Low	Low
3-nop	MCR enzyme inhibition	Established	Deployable^1^	20–40% (up to 82%)	Low	Moderate	Limited evidence
Dietary concentrate increase	Shift to propionate fermentation; pH reduction	Established	Deployable	10–30%	Moderate (acidosis risk)	Low	Low
Nitrate supplementation	Alternative electron acceptor	Established	Deployable^2^	10–50%	High (toxicity risk)	Low	Low
Tannis/essential oil	Direct inhibition; protozoal suppression	Developing	Pilot stage	0–30%	Moderate (digestibility)	Low	Variable
Fermented feeds	Enhanced propionate producers	Developing	Deployable^3^	5–15%	Low	Low	Low
Defaunation	Removal of H₂-producing protozoa	Developing	Research stage	~11%	High (fiber digestion)	Low	High
Vaccination	Immune targeting of methanogens	Experimental	Research only	0–10%	Unknown	High	High
Gene-edited forages	Enhanced lipids/secondary metabolites	Experimental	Research only	3.5–5.6% per 1% lipid increase	Unknown	High	Unknown
Gene-edited methanogens	Direct genetic modification	Experimental	Research only	Unknown	Unknown	Very High	High
Probiotics/acetogens	Alternative H₂ sinks	Developing	Pilot stage	Variable	Low	Moderate	High
Precision livestock farming	Monitoring and targeted management	Developing	Pilot stage	Variable	Low	Low	Low

^1^Regulatory approval obtained in some regions (Brazil, Chile) and under review in others (39). ^2^Requires careful management due to toxicity risk; professional supervision is recommended (43).^3^Particularly applicable to smallholder and mixed crop-livestock systems (23).

**Figure 4:**
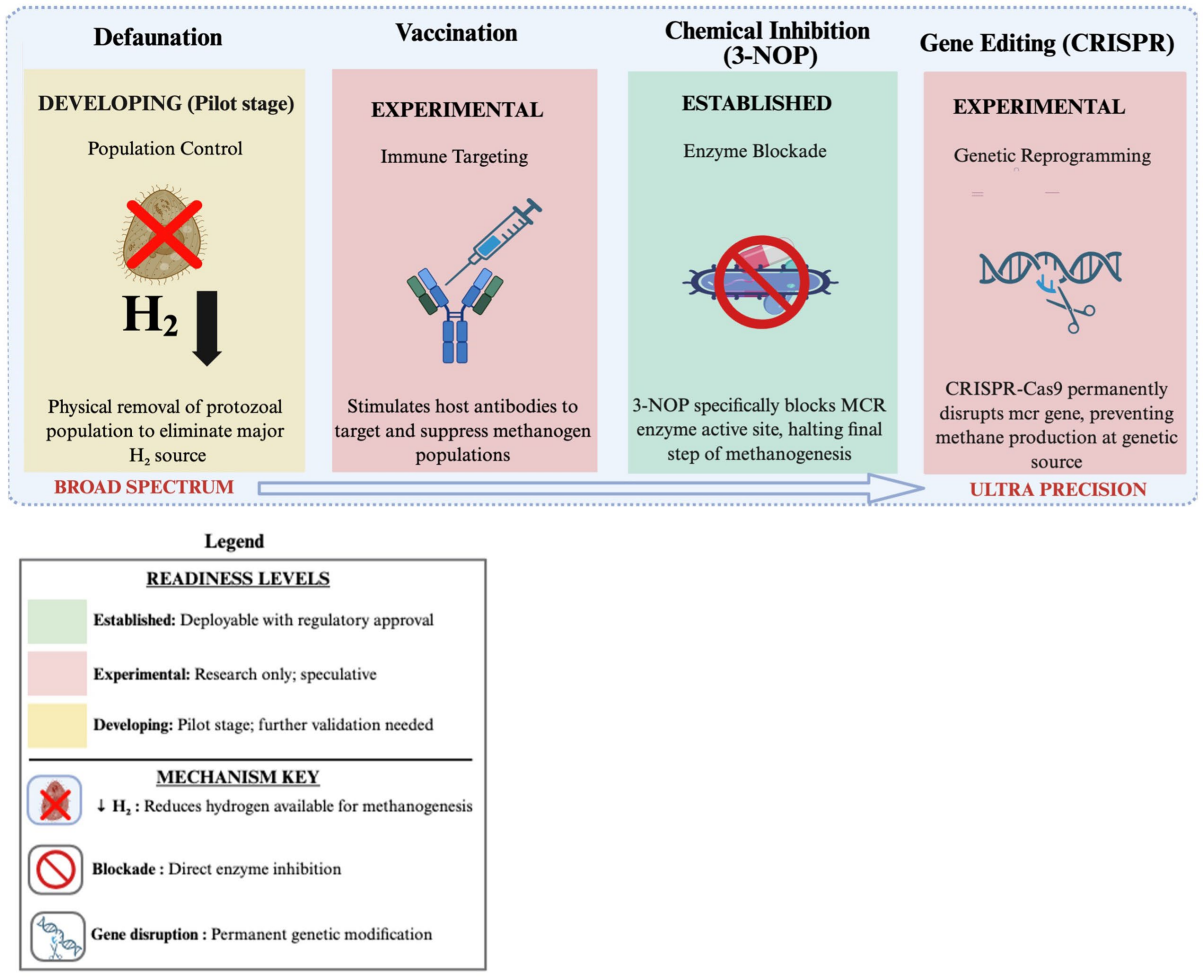
Overview of intervention strategies targeting ruminal methanogenesis across different levels of biological organization and readiness. The schematic summarizes approaches ranging from microbial community-level interventions (defaunation, competitive exclusion) to molecular-scale strategies (enzyme inhibition, gene editing). Readiness levels are indicated by background color: Green, Established (deployable with regulatory approval); Yellow, Developing (pilot stage, further validation needed); Red, Experimental (research only, speculative applications requiring substantial development). Created in BioRender. Kiadii, O. (2026) https://BioRender.com/y4vecnd.

A comparative overview of intervention strategies targeting ruminal methanogenesis, organized by biological scale and readiness level for practical implementation, is provided in Figure 4.

### 5.1 Established and deployable strategies

#### 5.1.1 Lipid supplementation

Dietary lipid inclusion is among the most thoroughly researched mitigation strategies (34). Lipids act through multiple mechanisms: direct inhibition of methanogenic archaea (particularly medium-chain saturated fatty acids), suppression of protozoa that facilitate interspecies hydrogen transfer (unsaturated fatty acids), and provision of an alternative hydrogen sink through biohydrogenation (35, 36). Meta-analyses indicate average methane reductions of approximately 15% with lipid supplementation, though efficacy varies substantially with lipid type, inclusion rate, and basal diet composition (37). Excessive lipid inclusion (>6–8% of dietary DM) may compromise fiber digestibility, reduce dry matter intake, and alter milk fat composition, necessitating careful formulation (35).

#### 5.1.2 3-Nitrooxypropanol (3-NOP)

3-NOP is a synthetic inhibitor of methyl-coenzyme M reductase (MCR), the enzyme catalyzing the final step of methanogenesis (38). By directly reducing methanogenic hydrogen capture ( $k_{m}$ ) in the conceptual framework, 3-NOP redirects hydrogen toward alternative sinks such as propionate formation. Meta-analyses report average methane reductions of approximately 30%, with some studies demonstrating reductions up to 82% under specific conditions (39). Importantly, 3-NOP has demonstrated efficacy without compromising dry matter intake, milk production, or growth performance in most studies (29). Regulatory approvals have been obtained in several regions, though cost and supply chain considerations influence adoption (40).

#### 5.1.3 Dietary carbohydrate manipulation

Shifting from high-forage to high-concentrate diets generally reduces methane yield (g CH₄/kg DMI) by promoting propionate production and lowering ruminal pH (41). However, this strategy must be balanced against animal health considerations (particularly subacute ruminal acidosis risk), feed costs, and system-level sustainability assessments. The magnitude of methane reduction varies with carbohydrate type; starch-based concentrates typically produce greater reductions than digestible fiber sources (42).

#### 5.1.4 Nitrate supplementation

Nitrate serves as an alternative electron acceptor, competing with CO₂ for reducing equivalents and redirecting hydrogen flow away from methanogenesis (7). Reported methane reductions range from 10–50% depending on dose and adaptation period. However, nitrate supplementation carries toxicity risks (methemoglobinemia) that require careful management, limiting widespread adoption in some production systems (43).

### 5.2 Developing and context-dependent strategies

#### 5.2.1 Tannins and essential oils

Plant secondary metabolites, including condensed and hydrolyzable tannins, saponins, and essential oils, exhibit anti-methanogenic activity through direct inhibition of methanogens, suppression of protozoa, or modulation of fermentation pathways (44). Reported efficacy varies widely (0–30% reduction) depending on compound source, dose, and basal diet. Higher doses may compromise fiber digestibility and protein utilization, creating trade-offs between methane reduction and animal performance (45). The variable concentration of bioactive compounds in natural sources presents additional challenges for consistent application (46).

#### 5.2.2 Fermented feeds

Bio-fermented crop residues and silages can alter rumen microbial communities in favor of propionate-producing bacteria, enhancing alternative hydrogen sink capacity (5, 47). These strategies are particularly relevant in smallholder and mixed crop-livestock systems where fermented feeds are already integrated into production practices. Reported methane reductions are modest (5–15%) but may be achieved alongside improvements in nutrient digestibility and animal performance (23).

#### 5.2.3 Defaunation

Removal of rumen protozoa reduces methane emissions by approximately 11% on average by eliminating a major hydrogen source and disrupting protozoal-methanogen symbiosis (48, 49). However, protozoa contribute to fiber degradation, microbial protein recycling, and fermentation stability (50). Defaunation may impair fiber digestibility and alter nitrogen metabolism, and maintaining protozoa-free animals is difficult under practical production conditions (14). Current research emphasizes modulation rather than elimination of protozoal activity.

### 5.3 Experimental and emerging strategies

#### 5.3.1 Vaccination

Vaccination strategies aim to stimulate host immune responses against methanogenic archaea, reducing methanogen populations or activity (51). While conceptually attractive due to potential for herd-wide application, methane vaccines have yielded inconsistent results in experimental trials (52). Challenges include microbial redundancy (multiple methanogen species and strains), antigenic variation, and the difficulty of generating sustained immune responses in the rumen environment. Vaccination remains an experimental approach requiring substantial further development before practical application can be considered (11).

#### 5.3.2 Gene editing of forage crops

CRISPR-mediated enhancement of lipid biosynthesis or secondary metabolite production in forage crops could theoretically develop naturally anti-methanogenic pastures (53). Increasing dietary lipid content by 1% is associated with methane reductions of approximately 3.5–5.6% (54, 55). However, scalability depends on regulatory approval, consumer acceptance, seed distribution infrastructure, and demonstration of consistent efficacy across environments and management systems.

#### 5.3.3 Gene editing of rumen methanogens

Direct genetic modification of methanogenic archaea using CRISPR-based approaches has been demonstrated in laboratory settings, with successful knockdown of key methanogenesis genes, including *mcr* (53). However, substantial challenges remain delivery efficiency in complex microbial communities, strain-specific responses, ecological stability of modified populations, potential for horizontal gene transfer, and regulatory/ethical considerations (56). This approach remains firmly in the experimental research domain with no near-term practical applications anticipated.

### 5.4 Strategic enhancement of alternative hydrogen sinks

Rather than solely suppressing hydrogen production or methanogenesis, effective mitigation can emphasize enhancing competitive sinks for metabolic hydrogen. Propionate formation and reductive acetogenesis serve as beneficial hydrogen sinks that convert H₂ into metabolically useful products (57). Feed formulation strategies that balance fermentable carbohydrates can encourage propionate-producing bacteria such as *Megasphaera elsdenii* and *Selenomonas ruminantium* (58). From a production standpoint, promoting propionate not only reduces methane emissions but also improves glucose supply through hepatic gluconeogenesis, directly supporting milk synthesis and growth performance (57). This dual advantage strengthens the conceptual basis for hydrogen redirection strategies compared to purely suppressive interventions.

### 5.5 Critical trade-off, economic feasibility, and long-term stability

The scalability of methane mitigation strategies ultimately depends on ecological stability, productivity preservation, and economic feasibility under commercial production conditions (59). Several critical considerations warrant explicit attention:

#### 5.5.1 Productivity trade-offs

Many interventions that reduce methane emissions may also affect nutrient digestibility, feed intake, or productive performance. For example, tannin supplementation at doses sufficient for methane reduction (>2–3% of DM) has been associated with reduced neutral detergent fiber and crude protein digestibility in some studies, potentially compromising animal performance (45). Similarly, excessive lipid supplementation can reduce dry matter intake and alter milk fat composition (35). Methane reduction does not always coincide with improved productivity, and trade-offs must be evaluated on a case-by-case basis.

#### 5.5.2 Microbial adaptation

Rumen microbial communities exhibit functional redundancy and adaptive capacity that may partially compensate for interventions over time (7). Evidence for adaptation to 3-NOP is limited in short- and medium-term studies, but longer-term data across multiple production cycles remain sparse (29). Ionophore efficacy for methane reduction has been observed to diminish over time in some studies, though mechanisms remain incompletely characterized (60). Monitoring for adaptation should be integrated into long-term mitigation programs.

#### 5.5.3 Economic feasibility

Adoption of mitigation strategies depends critically on economic viability. Costs associated with feed additives, genetic selection, and precision technologies must be weighed against productivity gains and reductions in methane intensity. Strategies that simultaneously enhance feed efficiency and lower emissions intensity are more likely to achieve sustainable adoption than those imposing net costs on producers (61). Economic feasibility varies substantially across farming systems, regions, and production intensities, necessitating context-specific evaluation.

#### 5.5.4 System compatibility

Mitigation strategies must align with existing herd management practices, infrastructure, and labor constraints. For example, daily feed additive administration is more feasible in confinement feeding systems than in extensive grazing operations. Strategies requiring specialized equipment or technical expertise may face adoption barriers in resource-limited settings (62).

## 6 Discussion

### 6.1 A framework for integrated mitigation strategies

The complexity and resilience of the rumen ecosystem preclude single-solution approaches to methane mitigation. The conceptual frameworks developed in this review suggest that lasting reductions in methane emissions will require integrated strategies that simultaneously address microbial ecology, hydrogen flow, and host productivity. Based on the genetic-microbiome co-evolution framework and the conceptual fermentation kinetics framework, we propose that coordinated management across three interconnected domains may offer advantages over isolated interventions.

#### 6.1.1 Genetic selection as a foundational layer

Incorporating traits associated with low methane emissions and high feed efficiency into existing breeding programs may provide cumulative, long-term benefits. Microbial biomarkers, such as reduced *mcrA* gene abundance or favorable archaeal-to-bacterial ratios, have been proposed as selection criteria, though validation in diverse populations and production environments remains necessary (11, 14). Building a genetically predisposed herd could reduce dependence on ongoing downstream interventions and ensure that mitigation improvements are retained across generations. However, the practical implementation of microbiome-informed breeding faces substantial challenges, including the cost of microbiome profiling, genotype-by-environment interactions, and the need to balance methane-related traits with other economically important production characteristics (32).

#### 6.1.2 Nutritional modulation for dynamic hydrogen management

Dietary strategies that actively influence hydrogen flow can complement genetic predisposition. Inhibitors such as 3-NOP produce rapid and substantial methane reductions by directly targeting methanogenesis (29, 44). Supporting additives, including tannins and selected lipid sources, can reduce protozoal populations and indirectly inhibit methanogens while promoting alternative hydrogen sinks (35, 44). When combined strategically, these additives may produce additive or synergistic effects, potentially allowing lower inclusion rates and reducing risks to fiber digestion and animal performance. However, systematic evaluation of combination strategies remains limited.

#### 6.1.3 Precision microbial manipulation for ecosystem steering

Emerging approaches, including next-generation vaccines, targeted bacteriophages, and engineered hydrogenotrophic bacteria, may eventually enable more precise manipulation of rumen microbial networks (51, 56). CRISPR-based editing of forage crops represents a longer-term strategy for modifying hydrogen metabolism at the feed source (53). These interventions operationalize the conceptual fermentation kinetics framework by enabling intentional redirection of hydrogen flow, though substantial hurdles in research, regulatory acceptance, and societal acceptance remain before practical application.

### 6.2 Multi-omics research priorities

Realizing the potential of integrated mitigation strategies requires robust multi-omics frameworks that capture both short-term effectiveness and long-term system stability. Longitudinal studies are needed to evaluate the persistence, ecological impacts, and unintended consequences of introducing novel interventions into ruminant systems (11, 63).

Metagenomic approaches should focus on monitoring the stability of targeted traits, potential horizontal gene transfer, and population dynamics of key taxa involved in hydrogen redirection, including propionate-producing *Succinivibrionaceae* and acetogenic *Lachnospiraceae* (57). Metatranscriptomics can provide functional validation by confirming sustained changes in gene expression patterns, including repression of methanogenesis-related genes (e.g., *mcr*) and upregulation of genes involved in alternative hydrogen sinks, such as fumarate reductase in the propionate pathway (5, 16). Metabolomics can verify that desired fermentation end-products, such as increased propionate: acetate ratios, are maintained without compromising host health or productivity (11, 53).

A critical translational priority is the identification of functional biomarkers that can indicate hydrogen redirection status in real time. The ratio of transcripts encoding hydrogen-producing enzymes (e.g., [FeFe]-hydrogenases) to hydrogen-consuming enzymes (e.g., fumarate reductase) has been proposed as a dynamic indicator of the rumen hydrogen economy state (5, 16). Such functional metrics move beyond taxonomic descriptions to capture the metabolic state of the microbiome, potentially enabling more precise management tailored to individual animals and production systems (11).

Finally, advancement from descriptive multi-omics to predictive systems biology represents a necessary paradigm shift. Integrating host genetics, microbial community dynamics, and fermentation kinetics into mechanistic computational models would enable in silico hypothesis testing before field deployment. Such “virtual rumen” platforms, while still in early development, could accelerate innovation, reduce experimental costs, and support rational design of mitigation strategies (11, 57).

### 6.3 Experimental approaches to validate framework predictions

To move beyond conceptual development toward empirical validation, we propose three experimental designs that could test core predictions of the frameworks presented in this review:

#### 6.3.1 Design 1: genetic selection validation study

A multi-generational selection experiment using divergent selection for archaeal: bacterial ratio or *mcrA* transcript abundance. Hypotheses to be tested include: (i) selection for low methanogen abundance produces heritable changes in offspring microbiome composition; (ii) selected lines differ in methane yield under standardized dietary conditions; and (iii) selection response is stable across dietary contexts. Design would require: ≥100 animals per line, controlled mating, standardized phenotyping protocols, and longitudinal microbiome and methane measurements across multiple parities and diets.

#### 6.3.2 Design 2: dietary hydrogen challenge study

A factorial experiment systematically varying hydrogen production ( $R_{\text{prod}}$ ) through forage: concentrate ratio (3 levels) and hydrogen sink capacity ( $\sum R_{\text{sink},i}$ ) through propionate enhancers (e.g., 3-NOP, nitrate; 3 levels including control). Hypotheses: (i) methane production increases with ( $R_{\text{prod}}$ ) when $\sum R_{\text{sink},i}\;$ is constrained; (ii) interventions enhancing $\sum R_{\text{sink},i}$ show greater efficacy under high $R_{\text{prod}}$ conditions; and (iii) interaction effects can be interpreted through the hydrogen partitioning framework. Design would require a respiration chamber or GreenFeed measurements, rumen fluid sampling for VFA and hydrogen metrics, and microbiome profiling.

#### 6.3.3 Design 3: integrated systems trial

A herd-scale evaluation combining genetic selection (using sires with divergent methane breeding values), precision feeding (phase-specific diets), and real-time monitoring (GreenFeed or sniffers) under commercial conditions. Hypotheses: (i) combined genetic and nutritional strategies produce additive or synergistic methane reductions; (ii) individual animal variation in response can be partially explained by baseline microbiome features; and (iii) mitigation benefits persist across production cycles. Design would require a multi-year commitment, commercial herd access, and a comprehensive data collection infrastructure.

### 6.4 Practical considerations for livestock production systems

Translating the conceptual management of the rumen hydrogen economy into commercial livestock systems requires a structured, phased approach. It is important to emphasize that the following considerations represent potential pathways for implementation rather than prescriptive recommendations; substantial validation work remains before these frameworks can guide routine on-farm decision-making.

#### 6.4.1 Breeding and genetic integration

Producers may consider incorporating methane-related traits into existing genetic improvement programs where selection indices and data infrastructure permit. When available, microbial biomarkers could supplement traditional selection criteria targeting milk yield, growth rate, or carcass quality. Selecting animals that demonstrate better feed conversion efficiency and lower methane emissions per unit of product may provide cumulative, long-term mitigation benefits. However, inclusion of methane-related traits in breeding goals should be balanced against other economically important characteristics, and the cost-effectiveness of microbiome profiling for commercial breeding programs has not been established.

#### 6.4.2 Precision feed formulation

Feed formulation may be guided by hydrogen redirection principles rather than methane suppression alone. Practical nutritional considerations include: optimizing forage-to-concentrate ratios to encourage propionate production while maintaining rumen health; strategic use of approved additives such as 3-NOP where economically feasible and regulatory approved; controlled inclusion of unsaturated lipid sources to suppress protozoal-mediated hydrogen transfer while preserving fiber digestibility; and careful administration of nitrate supplements under professional supervision to ensure animal safety. Dietary interventions should be tailored to production stage (early lactation, finishing phase, growing animals) to avoid compromising energy balance or rumen health.

#### 6.4.3 Monitoring and adaptive management

Routine monitoring can help maintain mitigation strategy effectiveness over time. Producers may track feed conversion ratio, milk production, average daily gain, and (where measurement technology permits) methane intensity. Monitoring rumen health indicators such as milk fat percentage, manure consistency, and incidence of subacute ruminal acidosis can help detect unintended consequences of dietary interventions. Periodic assessment of additive effectiveness may identify possible microbial adaptation.

#### 6.4.4 Economic feasibility and implementation roadmap

Successful adoption depends on economic viability, which must be evaluated on a farm-by-farm basis. Producers should conduct cost–benefit assessments comparing additive costs against productivity gains, genetic investment against long-term emission reduction potential, and feed reformulation costs against improved feed efficiency. A phased implementation approach may include: (i) short-term: improved diet formulation and approved additives under professional supervision; (ii) medium-term: incorporation of methane-related traits into breeding goals where selection infrastructure exists; and (iii) long-term: implementation of emerging precision technologies as regulatory and economic conditions permit.

It is essential to acknowledge that these frameworks are complementary to, rather than a replacement for, diverse agricultural, economic, and technological approaches to sustainable livestock production. Predictive management of the rumen hydrogen economy remains a developing research area, and current capacity for *in vivo* prediction is limited. Economic feasibility varies substantially across farming systems, and methane reduction does not always coincide with improved productivity in all contexts. Mitigation strategies that lower methane intensity per unit of product while maintaining or enhancing profitability are most likely to achieve sustainable adoption.

### 6.5 Implications for global sustainable agriculture

The conceptual frameworks outlined in this review have potential implications for global sustainable agriculture, though their practical impact depends on successful validation and implementation. By treating enteric methane as a dynamic and potentially manageable component of the rumen hydrogen economy, this approach may contribute to achieving international mitigation targets, including those articulated in the Global Methane Pledge, without necessarily compromising animal productivity or farmer livelihoods (4). Rather than relying on isolated, short-term solutions, the frameworks encourage coordinated consideration of multiple biological levels from host genetics and microbial communities to fermentation processes and dietary inputs. Leveraging cumulative genetic improvements and enhanced feed efficiency may help decouple productivity from emissions intensity, delivering environmental benefits alongside economic viability. This systems perspective positions methane mitigation as an opportunity for innovation in climate-smart food systems rather than solely a constraint on productivity (61).

## 7 Conclusion

Enteric methane mitigation in ruminant systems may benefit from shifting attention from isolated intervention strategies toward integrated, system-level consideration of rumen metabolism. This review has synthesized evidence indicating that methane production is best understood as an emergent outcome of hydrogen distribution within the rumen microbial network, rather than as a fixed endpoint of fermentation.

By introducing the genetic-microbiome co-evolution framework alongside a conceptual fermentation kinetics perspective, this work offers a unified conceptual basis for linking host genetics, microbial ecology, and nutritional management to methane outcomes. These frameworks are presented as hypothesis-generating tools that may enable a transition from descriptive observation toward mechanistic interpretation of hydrogen flow dynamics. It is essential to emphasize that substantial validation work remains before these conceptual frameworks can guide routine on-farm decision-making.

In practice, this approach may inform the development of precision systems that integrate genetic selection, targeted feeding strategies, and emerging biotechnological tools to improve feed efficiency while reducing emissions intensity. Strategies that enhance the productive utilization of hydrogen, particularly through propionate formation, offer potential dual benefits by improving animal performance while reducing energy losses associated with methane formation. However, context-dependent trade-offs must be carefully evaluated, and methane reduction does not universally coincide with productivity improvements.

Future progress will require integration of multi-omics data with mechanistic models to validate the long-term stability, scalability, and economic feasibility of hydrogen-centered mitigation strategies under commercial conditions. Advancing toward predictive and system-based understanding of the rumen hydrogen economy positions methane mitigation as a catalyst for innovation in climate-smart livestock production rather than solely as an environmental compliance obligation. The conceptual frameworks presented here provide a foundation for this advancement, while acknowledging the substantial empirical work that remains necessary to realize their practical potential.
